# Ultrasonic-assisted enzymatic extraction of silymarin from the Silybum marianum seed shell and evaluation of its antioxidant activity in vitro

**DOI:** 10.17179/excli2015-257

**Published:** 2015-07-27

**Authors:** Fei Zhao, XinHua Li

**Affiliations:** 1 College of Food Science, Shenyang Agricultural University, Shenyang 110866, China

**Keywords:** Silybum marianum, ultrasonic assisted enzymatic extraction, Silymarin, Box-Behnken design, SEM, antioxidant activity

## Abstract

This study revealed the optimal conditions for the Ultrasonic-Assisted Enzymatic Extraction (UAEE) of silymarin, and include: the concentration of ethanol, 50 %; enzyme concentration, 30 U/mg; liquid-solid ratio, 6:1; an extraction time of 120 min; and the ultrasonic power at 180 W. The extraction rate was 7.86 %, which is higher, by 74.67 %, than that of the silymarin extract from the Silybum marianum meal prepared by a distinct approach. SEM micrographs of the inner and outer surfaces of the Silybum marianum shell obtained by variant extractions demonstrated that the extraction of silymarin required the destruction of cell walls. The results suggest that UAEE is a promising alternative for the extraction of silymarin. The antioxidant activities of the silymarin were evaluated in vitro by its capabilities to scavenger the DPPH, hydroxyl and superoxide free radicals, as well as by its tyrosinase inhibitory activity. The results showed that silymarin has significant antioxidant activity, thus it can be used as a functional food material against oxidative stress. We believe that the knowledge gained from this study should contribute to the further development and application of this resource.

## Introduction

Silymarin, derived from the Silybum marianum plant, has been widely used for centuries for its hepato-protective properties. It has also been used against toxic liver damage, hepatitis and cirrhosis (Mayer et al., 2005[18]; Wellington and Jarvis, 2001[27]; Saller et al., 2001[23]; Křen et al., 2005[14]). In addition to its antioxidant properties, it has been reported to have exceptional anti-tumor promoting activity (Singh and Agarwal, 2004[24]; Davis-Searles et al., 2005[7]) and has also been linked to the prevention of skin cancer (Katiyar, 2003[11]). Silymarin consists primarily of a mixture of active flavonolignans isomers: silychristin, Sc; silydianin, Sd; and two groups of diastereoisomers, silybins A and B (Sb A, Sb B), and isosilybins A and B (ISb A, ISb B) (Kim et al., 2003[12]; Lee and Liu, 2003[17]). The isomers of silymarin have been found to have different biological activities (Křen et al., 2000[13]; Chlopcíková et al., 2004[6]). 

Several methods for the extraction of silymarin from the seeds of Silybum marianum have been described (Alvarez et al., 2003[2]). Previously used extraction methods include methanol Soxhlet extraction, pressurised liquid extraction, and reflux and mechanical shaking extraction using (hot/liquid) water as the solvent. The extraction yield of silymarin from the seeds of Silybum marianum using either ethanol, methanol, acetonitrile or acetone as extraction solvents have been investigated (Wallace et al., 2003[25]). However, the most important features of ultrasonic assisted enzymatic extraction (UAEE) have not yet been fully elucidated and the establishment of the parameters of the silymarin UAEE process requires the systematic analysis of the experimental data.

The silymarin in Silybum marianum is mainly found wrapped in the cell wall, composed mainly of cellulose, but with the ethanol extraction method the cell wall cannot be completely broken for the dissolution silymarin; thus, there is considerable resistance, resulting in a greatly affected and wasteful extraction that is more costly and yields an extract with impurities and of not high quality.

Response surface methodology (RSM) is a useful package of statistical and mathematical techniques used for developing and improving processes by solving simulation-based optimization problems, which was first introduced by Box and Wilson. One of the advantages of this method is its ability to take into account the interactions among different variables as opposed to the traditional one variable at a time analysis. Additionally, as an experimental design, RSM can reduce the number of experiments and provide a mathematical model. (Guan and Yao, 2008[8]; Karacabey and Mazza, 2010[10]; Nagendra et al., 2011[21]; Wang et al., 2012[26]). Hence, RSM can be effectively used to evaluate the effects of multiple factors and their interaction on one or more response variables (Myers and Montgomery, 2002[19]) and is thus applicable to the silymarin extraction problems.

In the present study, the isolation of the silymarin from the Silybum marianum seed shell was investigated and the operational parameters were optimized using a central composite rotatable design combined with RSM. The seed shell silymarin extract processed by the UAEE was also compared with the silymarin extract from the Silybum marianum meal prepared by heating extraction with ethanol. To verify that the silymarin yield and the quality attained by UAEE was improved over that achieved by heating extraction, changes and composition were monitored by scanning electron microscopy (SEM) and high pressure liquid chromatography (HPLC) analysis, respectively. In addition, the antioxidant activity of the silymarin from Silybum marianum seed shell was also determined in vitro.

## Materials and Methods

### Materials and instruments

The Silybum marianum seeds were obtained from Panjin Tianyuan Pharmaceutical Co., Ltd. (Panjin, China); the cellulase (Huzhou Lilly Biotechnology Co., Ltd., city, country) used had an activity greater than 50,000 u/g. All chemicals and solvents used were of analytical grade. The reference compound Rutin was from the Chinese Institute for the Control of Pharmaceutical and Biological Products (Beijing, China). Water was deionized and purified by a Milli-Q water purification system (Millipore, Bedford, MA, USA). DPPH, ethylene diamine tetraacetic acid (EDTA), H_2_O_2_, and ascorbic acid were from Sigma Chemical Co. (St. Louis, MO, USA). Sodium dihydrogen phosphate, disodium hydrogen phosphate, potassium ferricyanide and trichloroacetic acid were from the Hangzhou Reagent Company (Hangzhou, PR China). The DK-S24 thermostatic water bath (Shanghai Yarong Biochemistry Instrument Factory, Shanghai, China) was used for heating the extraction of silymarin; a KQ2200DB ultrasonic cleaner (Kunshan Ultrasonic Instrument Co., Ltd) was used for sonication; the UV-1700 spectrophotometer (Shimadzu Corporation, Japan) was used for analysis of total silymarin; the RE-52AA rotary evaporator (Shanghai Yarong Biochemistry Instrument Factory, Shanghai, China) was used for concentrating the samples; a FD-3 freeze drier (Beijing Boyikang Experimental Instrument CO., Ltd., Shanghai, China) was used to dry the concentrated sample. The chromatography grade methanol and formic acid used for HPLC were from Dima Technology Inc. (Shenyang, China).

### Extraction of silymarin

#### Extraction of silymarin from Silybum marianum meal

The dried powder of the Silybum marianum meal (500 g) was mixed in a beaker, at a liquid-solid ratio of 6:1, with 70 % ethanol. Subsequently, extraction was performed in a water bath for 24 hours; and then the total silymarin content was determined to calculate the extraction rate.

#### The UAEE of silymarin from Silybum mariannum seed shell

After rolling peeling, the Silybum marianum fruit was mixed with water; the seed coat was next isolated by winnowing, and then collected, dried and crushed. Afterward the powder was passed through a 40 mesh sieve and set aside. To extract the silymarin,1 kg of dried powder from the Silybum marianum seed coat was mixed with petroleum ether (boiling range 60 - 90 °C), skimmed for 8 h, filtered, and then defatted for 4 h with petroleum ether. An amount of 500 g of dry weight, nonfat, Silybum marianum seed coat powder was weighed and dissolved in 100 mL of 50 % ethanol. The beaker with this mixture was placed in a water bath kept at a temperature of 35 °C, the pH was adjusted to 5.0, the solution was then sonicated with an ultrasonic output power of 200 W for the duration of the extraction, and the digestion with 3 U/g of the enzyme was carried out for 1h; after which the enzyme was inactivated by incubation at 85 °C for 10 min. Subsequently, the solution was filtered by vacuum suction filtration and each filtrate (about 250 mL) was the concentrated using the rotary evaporator until it reached 250 mL of solution and lastly the total flavonoid content was determined.

### Determination of total silymarin

The total content of silymarin was determined by the colorimetric method (Aliakbarian et al., 2012[1]; Antoine et al., 2004[3]; Karabegovic et al., 2011[9]; Zhang et al., 2011[28]). We accurately weighed appropriate amount of silybin (accurate to 0.1 mg), formulated as a stock solution in methanol containing silybin and isosilybin. The stock standard solution formulated as a mixed standard solution, and then diluted 5-fold with the mobile phase, 10 times, 20 times, 40 times, 50 times the standard series hybrid solution. The sample was measured by retention time peak area of qualitative, external standard method with peak area quantification. Respectively, according to chromatographic conditions were measured and five peak area, then the peak area of the vertical axis, standard injection volume as the abscissa, the standard curve Y = 14670X125024, R^2^ = 0.9996. (Where Y is the absorbance value of the sample, and X is the sample concentration).

### Reverse-Phase-HPLC analysis

**Figure 1:**
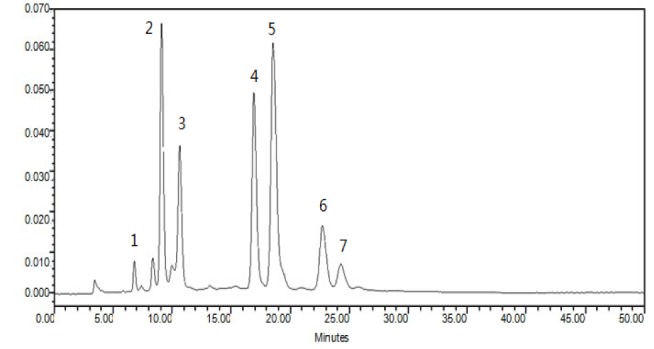
Typical chromatogram of the silymarin extract. 1: toxifolin, 2: silychristin, 3: silydianin, 4: silybin A, 5: silybin B, 6: isosilybin A, 7: isosilybin B

The HPLC was used on a Shimadzu LC-20AVP system with two LC-20AT solvent delivery units, an SPD-20A UV-vis detector, a CTO-10ASVP column oven (Shimadzu, Kyoto, Japan), a T2000P workstation (Shenyang, China) and a reversed-phase C18 column (250 × 4.6mm, 5μm, Diamodsil^TM^). The conditions for the HPLC detection of silymarin were as follows: solvent A, methanol; solvent B, water (1 ‰ formic acid); gradient (A%), initial 4 min 43 %, 4-25 min 43-70 %, 25-30 min 70 %, 30.01 min 43 %, 40 min, stop; flow rate, 1mL/min; injection volume, 10μL, wavelength, 288 nm; column temperature,40°C. A sample chromatogram from the extraction of silymarin is shown in Figure 1(Fig. 1).

### Response surface design of experiments

On the basis of a single factor experiment, according to Box-Behnken central composite design principles, the process for the optimal extraction of silymarin was evaluated using RSM based on a three-level five-factor Box Behnken design to determine the optimum extraction conditions including ethanol concentration, enzyme concentration, extraction time, liquid-solid ratio and ultrasonic output power. The experimental design factors levels are shown in Table 1(Tab. 1).

### Data analysis and statistical methods

The polynomial equation, the response surface curve and the contour plots were generated using the “Design-Expert” software (version 8.0, Stat-Ease Inc., Minneapolis, MN, USA). The experimental data were statistically analyzed using the “Design-Expert” package for analysis of variance (ANOVA). All experimental results were centered at using three parallel measurements of the mean ± SD. P values < 0.05 were regarded as significant and P values < 0.01 as very significant.

### Evaluation of the antioxidant activities of silymarin in vitro

#### Superoxide anion radical-scavenging activity

Measurement of superoxide anion activity is based on the method described previously with minor modifications. The PMS-NADH system for the generation of superoxide radicals contains 3 mL of Tris-HCl buffer (16 mm, pH 8.0), 338 μm of NADH (adenine dinucleotide), 72 μm of television (nitro blue tetrazolium) and 30 μm PMS (phenazine methosulphate). Varying concentrations of samples ranging from 25 to 400 μg/mL were added to the PMS-NADH radical scavenging systems. The reaction mixture was stirred at room temperature for 5 minutes before the blank absorbance is read 560 nm. Tris-HCl buffer was used in the control or blank instead of sample. The decreased absorbance of the reaction mixture indicated an increased in superoxide anion scavenging activity. The removal of superoxide radicals by Total Flavonoids from Persimmon Leaves (TFPL) can be calculated using the following equation: 

Scavenging effect (%) = (1-A_sample560_/A_control560_) × 100 %

#### DPPH radical-scavenging activity

The free radical-scavenging activity of the purified silymarin was measured by the DPPH method as previously described (Karacabey et al., 2010[10]; Guan andYao, 2008[8]) with some modifications. The 0.4 mmol/L solution of DPPH in 95 % ethanol was freshly prepared daily, and 2 mL of this solution, together with 2 mL of 95 % ethanol, were added to 1mL samples of the purified silymarin at different concentrations in water. The mixture was shaken vigorously and left to stand for 30 min in the dark, and the absorbance was then measured at 517 nm against a blank. The decreased absorbance of the reaction mixture indicated an increased in free radical-scavenging activity, which was analyzed from the graph of the plotted inhibition percentage against compound concentration. Ascorbic acid was used as positive controls. The experiments were performed in triplicate and the data averaged. The radical-scavenging capability of the DPPH was calculated by the following equation:

E (DPPH • ) = [1- (A_i_-A_j_) / A_0_] × 100 %

where A_0_ is the absorbance of the DPPH solution without test sample (2 mL DPPH + 2 mL of 95 % ethanol); Ai is the absorbance of the test sample mixed with the DPPH solution (2 mL sample + 2 mL DPPH) and Aj is the absorbance of the sample without the DPPH solution (2 mL sample + 2 mL of 95 % ethanol).

#### Hydroxyl radical-scavenging activity. 

The hydroxyl radical-scavenging activity of samples of TFPL was measured using a modified Smirnoff and Cumbes' method. Hydroxyl radicals were generated in a solution of 2 mM EDTA-Fe (0.5mL), 3 % H_2_O_2_ (1mL), and 360 μg/mL crocus in 4.5 mL sodium phosphate buffer (150 mM, pH 7.4). The samples at concentrations ranging from 25 to 400 μg/mL were incubated at 37 °C for 30 min and hydroxyl radicals were detected by monitoring absorbance at 520 nm. In the control, distilled water and sodium phosphate buffer were used instead of test sample and H_2_O_2 _respectively. All the experiments were performed in triplicate and the data averaged. The hydroxyl radical-scavenging capability was calculated using the following equation: 

Scavenging effect (%) = (1-A_sample520_/A_control520_) × 100 %

#### Tyrosinase inhibitory activity assay

Tyrosinase (TYR) inhibitory activity was measured according to a previously described method. First stocks of all test samples were made by dissolving them in dimethyl sulfoxide (DMSO) to a concentration of 1.0 mg/mL. Each of these stocks was further diluted in DMSO to various concentrations. Subsequently, 30 μL of each diluted test sample was added to 970 μL of phosphate buffer pH 6.8 in a test tube and was then combined with 1.0 mL of L- tyrosine and 1.0 mL of mushroom TYR (200 units/mL). The resulting 3 mL test samples were incubated in a water bath at 37 °C for 20 minutes, and the enzyme activity was monitored by measuring the change in absorbance at 490 nm. Quercetin was used as reference. Each experiment was repeated at least twice. The percent inhibition of the TYR by the test sample was calculated as follows:

Tyrosinase activity inhibition rate ( %) = [1 - (C - D) / (A - B)] × 100 %

where A = A_490_- drug + enzyme, 

B = A_490_- drug - enzyme, 

C = A_490_ + drug + enzyme, 

D = A_490_ + drug - enzyme.

## Results and Discussion

### Single factor analysis method

The results in Table 2(Tab. 2) show that the yield of silymarin was markedly impacted by the different concentrations of ethanol. Extraction yield is the highest when 70 % ethanol is used as the extraction solvent. Below 70 % ethanol, the yield increased as the concentration of ethanol increased. In contrast, the yield decreased as the concentration of ethanol increased from 70 % to 90 %. In fact, the data in table 2(Tab. 2) indicated that there were significant differences among the yields obtained with 60, 80, 90 and 70 % ethanol (P < 0.05). Since the mean yield difference was the highest with about 70 %, the 70 % ethanol was selected as a center point for further RSM experiment.

Additionally, the results in Table 2(Tab. 2) revealed the striking effect that the different enzyme concentrations had on the yield of silymarin. When the enzyme concentration was under 30 %, the yields were improved by the increasing concentration of the enzyme. In contrast, when the enzyme concentration was above 30 %, the yields declined as the enzyme concentration increased from 30 to 50 %. Indeed, the results in Table 2(Tab. 2) show that there were significant differences among the yields attained with the 20, 40 and 30 % enzyme concentrations (P < 0.05). As the mean yield difference was the highest at an enzyme concentration of 30 %, the 30 % concentration was selected as the center point for further RSM experiment.

The results displayed in Table 2(Tab. 2) also demonstrated that the yield of silymarin increased greatly when the liquid-solid ratio grew from 2:1 to 6:1, and then it maintained a mild slope with increasing ratio of liquid to material. In effect, the data in Table 2(Tab. 2) also revealed the significant differences that existed among the 2:1, 4:1, and 6:1 ratios (P < 0.05); there was however no significant difference between the 6:1 and 8:1 ratios (P > 0.05). Thus, the liquid-solid ratio of 6:1 was selected as the center point for further RSM experiment.

Similarly, the results revealed that the yield of silymarin was clearly enhanced by the increase of ultrasonic power from 160 W to 240 W. Moreover, statistical analysis detected that there were significant differences among the ultrasonic powers tested (Table 2(Tab. 2)). However, it was also obvious from the mean ratio difference that the largest change in yield was caused from the ultrasonic power from 160 W to 180 W. Although the ultrasonic power 180 W was adopted for use in this work, after taking into account of extraction efficiency, it was not however selected as the center point for further RSM experiment. 

Additionally, the yield of silymarin was also markedly improved by the increase of the extraction time from 60min to 120 min. While, over 120 min, the yield decreased somewhat. This might be due to the decomposition of active compounds during the prolonged extraction time. The results in Table 2(Tab. 2) also revealed significant differences among the 60, 90 and 120 min reaction times (P < 0.05), but there were no significant differences among the 150 and 180 reaction times (P > 0.05). Therefore, the center point of extraction time chosen for RSM was 120 min.

### Diagnostic checking of the fitted model

According to the values obtained in the single factor experiment and the method of central composite design experiment, RSM was applied to monitor the extraction characteristics of the silymarin components in Silybum marianum and to determine the optimum conditions. Experiments were randomized as detailed in Table 3(Tab. 3).

Multiple regression analysis of the experimental data yielded the following second-order polynomial stepwise equation:

Y = 7.74-0.33A+0.044B-0.32C-0.16D+0.17AB+0.14AC-0.26AD+0.38BC+0.48BD-0.26CD-3.44A^2^-1.56B^2^-2.38C^2^-1.81D^2^

ANOVA was used to evaluate the model for significance and suitability, and a statistical summary was given in Table 4(Tab. 4). The model F value of 210.17 with a low probability P value indicates the high significance of the model. There was only a 0.01 % chance that a model F value so large could occur due to noise. The coefficient of determination (R^2^) was the proportion of variability in the data explained or accounted for by the model. The R^2^ of 0.9953 was therefore desirable. Previous studies (Le Man et al, 2010[16]; Chauhan and Gupta, 2004[4]) have emphasized the acceptance of any model with R^2^ > 0.75. Equally, the linear variables A and C, the quadratic variables and A^2^, B^2^, C^2^, D^2^ were statistically very significant at P < 0.0001; and the two-variable interactions AD, BC, BD and CD had significant influences (P < 0.05) on the extraction yield of silymarin, whereas the linear variable C and the two-variable interaction AB had no significant influence (P > 0.1) on the extraction yield of silymarin. By observing linear and quadratic coefficients, we concluded that the order of factors influencing the response value of the extraction yield of silymarin was as follows: ethanol concentration > extracting time > the liquid-solid ratio > enzyme concentration.

### Response surface analysis

**Figure 2:**
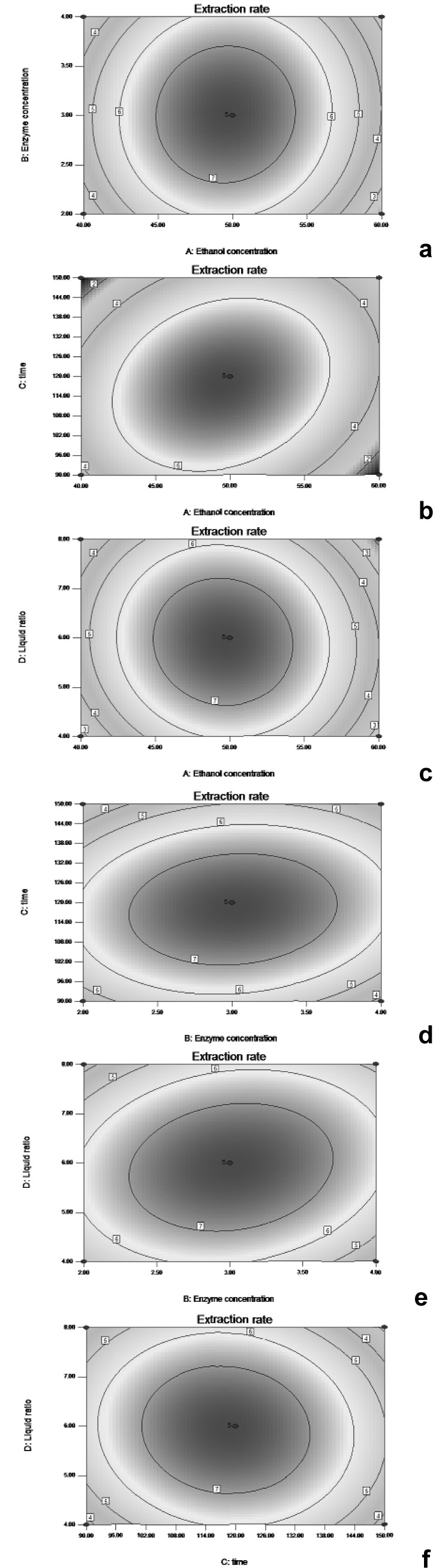
Contour plots (2D) showing the effect that the different extraction parameters (a: ethanol concentration; b: enzyme concentration; c: extraction time; d: liquid solid ratio) added on the response.

**Figure 3:**
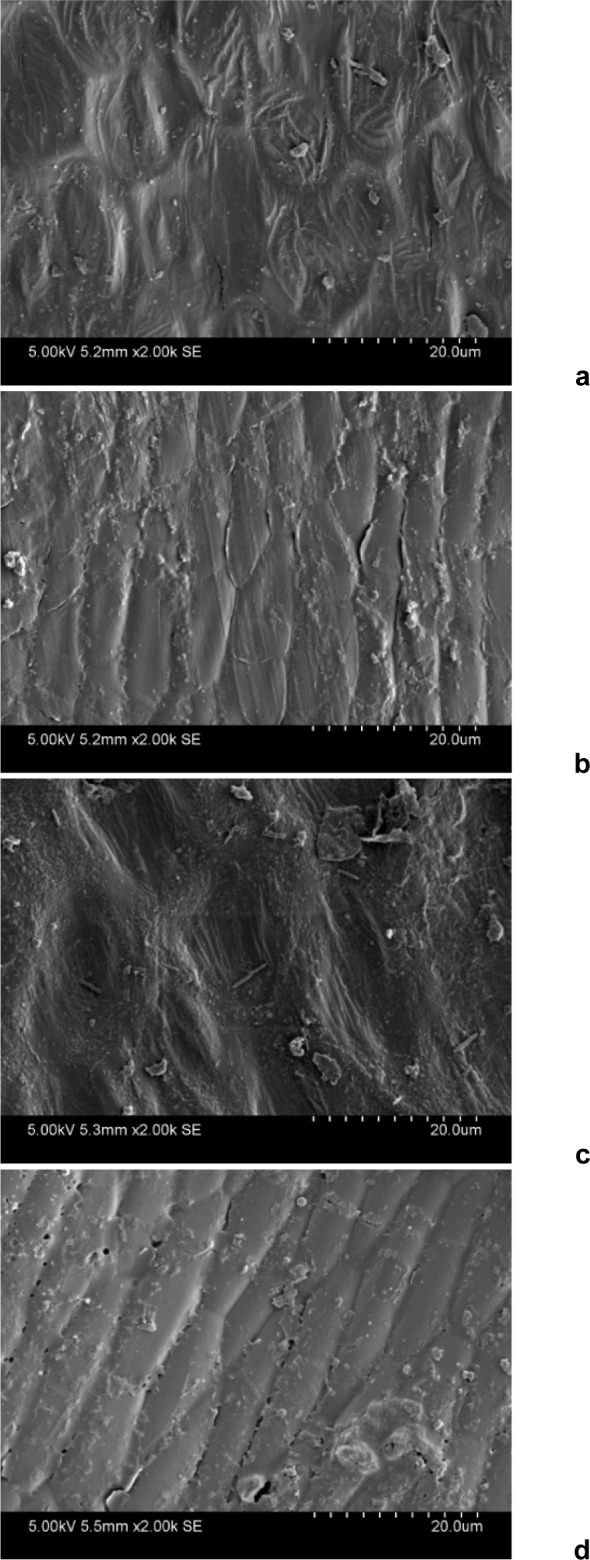
Scanning electron microscope of seed shells of Silybum marianum after different treatment processes: a: The inner surface of untreated Silybum marianum seed shells; b: The outer surface of untreated Silybum marianum seed shells; c: The inner surface of the UAEE-treated Silybum marianum seed shell; and d: The outer surface of the UAEE-treated Silybum marianum seed shell

Three-dimensional (3D) response surfaces and two-dimensional (2D) contour plots were used for the graphical representations of regression functions. They are presented in Figures 2(Fig. 2) and 3(Fig. 3) for the independent variables (enzyme concentration, ethanol concentration, extraction time, and liquid-solid ratio) and were obtained by keeping two of the variables constant, which indicated the changes in extraction yield under different conditions. 

**Figure 4:**
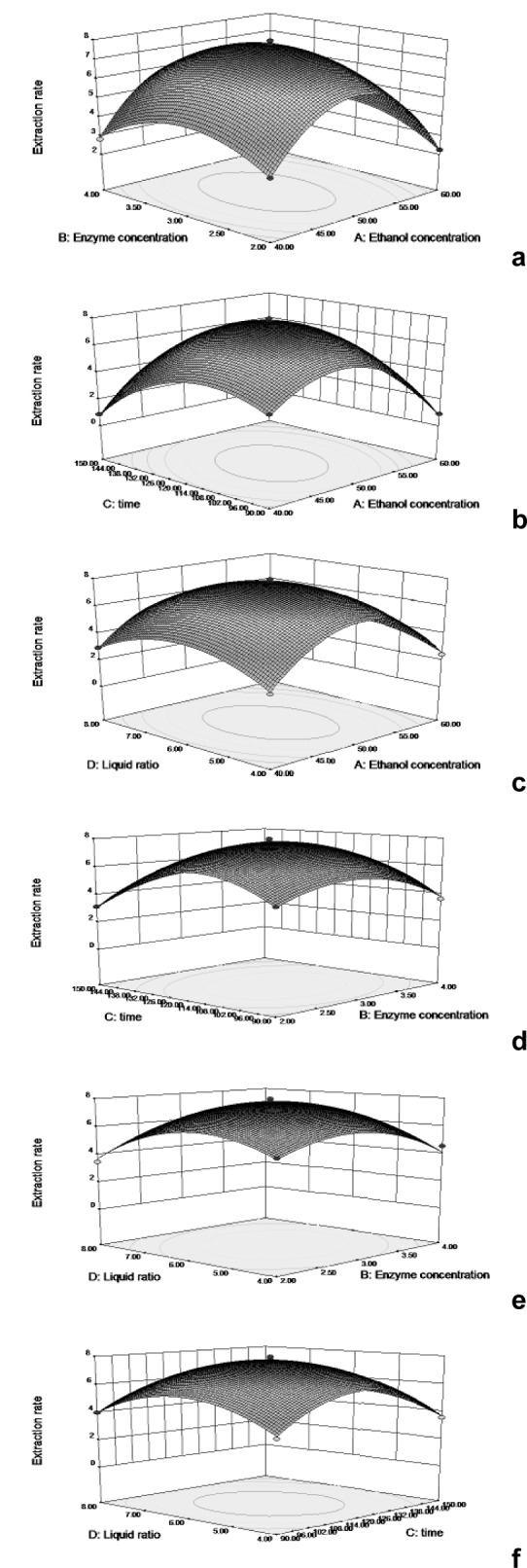
Response surface (3D) showing the effect that the different extraction parameters (a: ethanol con concentration; b: enzyme centration; c: extraction time; d: liquid solid ratio) added on the response.

Figures 2a(Fig. 2) and 4a(Fig. 4) show the 3D response surfaces and the elliptical contour plots, which illustrate the combined effect of ethanol concentration and enzyme concentration on the extraction yield. They revealed that at low and high levels of the ethanol and enzyme concentrations the extraction yield was minimal. When the ethanol concentration was at a certain value, the extraction yield increased with the increase of the liquid-solid ratio. On the other hand, the effect of the increase of the enzyme concentration on the extraction yield at a certain ethanol concentration was not significant.

The results shown in Figures 2b(Fig. 2) and 4b(Fig. 4), indicated that the mutual interactions between ethanol concentration and extraction time were significant when the other two variables were held constant. Similarly, as shown in Figures 2c(Fig. 2) and 4c(Fig. 4), the ethanol concentration and the liquid-solid ratio displayed a quadratic effect on the response, and the mutual interactions between them were also significant. In contrast, the combined effect of the extraction time and the enzyme concentration was not significant, as can be seen in Figures 2d(Fig. 2) and 4d(Fig. 4). The results presented in Figures 2e(Fig. 2) and 4e(Fig. 4) revealed that at a given enzyme concentration the surface was relatively flat, implying that the effect of the liquid-solid ratio on the rate of extraction of silymarin was not very evident. However, when the liquid-solid ratio was at a certain value, the rate of extraction of silymarin increased and then decreased. The effects seen in Figures 2f(Fig. 2) and 4f(Fig. 4) indicated that when extraction time was at a certain value, the extraction yield obviously increased with the liquid-solid ratio added. In addition, when the liquid-solid ratio was unchanged, the extraction yield increased as the extraction time increased. Overall, the response surface indicated that the extraction yield underwent noticeable increases depending upon the ethanol concentration, extraction time, and liquid-solid ratio, whereas no significant effect was observed in the enzyme concentration. This was in good agreement with our findings in the evaluation by ANOVA.

### Optimization and verification

 By using the Design Expert 8.0 software, the optimum conditions were determined and recognized as the practical optimum: ethanol concentration, 60 %; enzyme concentration, 30 U/mg; liquid-solid ratio, 6:1; and extraction time, 120 min. The estimated Y value, under those conditions, was 7.85 %; while the experimentally determined Y value was 8.1 %, which was consistent not only with the predictive values, but was also better than any single factor experiments. Therefore, the extraction conditions determined by RSM were not only accurate and reliable, but also have practical value by reflecting the expected optimization. In contrast to traditional techniques, this model takes into account the interactions among several independent variables. This work clearly shows that the extraction of silymarin from Silybum marianum can be improved by optimizing several key extraction parameters.

### Extraction explores various auxiliary mechanisms

In order to understand the mechanisms of the UAEE method and the inner and outer surfaces of the Silybum marianum seed shell, Scanning Electron Microscopy (SEM) was performed on the Silybum marianum seed shells with a table top scanning electron microscope (Hitachi T-l000; Hitachi High-Tech., Shanghai, China). The observations are depicted in the SEM microphaphs shown in Figure 3a-d(Fig. 3).

### Antioxidant activity analysis

#### Scavenging activity on DPPH radical

The DPPH contains a stable free radical, which has been widely used to assess the radical scavenging activity of antioxidants (Nagai et al., 2003[20]). The effect of antioxidants on the DPPH radical-scavenging activity was attributed to their hydrogen-donating ability (Chen et al., 2008[5]). 

**Figure 5:**
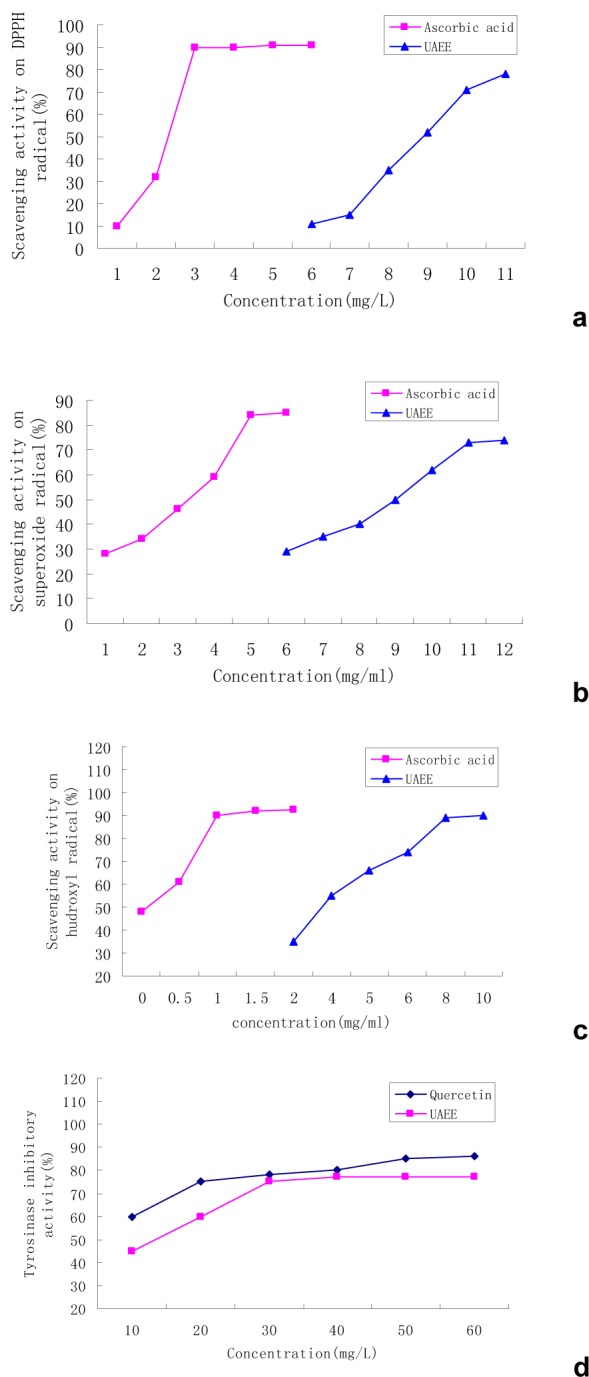
a: The DPPH radical-scavenging activity of silymarin, b: The superoxide radical-scavenging activity of silymarin, c: The hydroxyl radical-scavenging activity of silymarin, d: The tyrosinase inhibitory activity of silymarin

The results of the experiments to assess the DPPH radical-scavenging ability of purified silymarin are presented in Figure 5a(Fig. 5), and compared with ascorbic acid as control standards. The results showed that silymarin at concentrations ranging from 5-10 mg/L increased the radical scavenging activity on DPPH from 10 % to 74 %; which indicated that the purified silymarin has a significant concentration-dependent DPPH radical-scavenging activity.

#### Scavenging activity on hydroxyl radical

Hydroxyl radical, well known as the most reactive free radical, can react with almost all the biomolecules functioning in living cells in the form of abstracting hydrogen atoms, addition reactions and electron transportation (Lai et al., 2010[15]). The radical scavenging activity is not due to its direct scavenging but rather to inhibition of hydroxyl radical generation by chelating ions such as Fe^2+^ and Cu^2+ ^(Qi et al., 2006[22]). Moreover, hydroxyl radical can be generated by the reaction of Fe^2+^ and H_2_O_2_, and silymarin has Fe^2+^-chelating ability. Therefore, silymarin could reduce the generation of hydroxyl radical by chelating the Fe^2+^. The results in Figure 5b(Fig. 5) depict the measurement of the hydroxyl radical-scavenging activity of silymarin and indicated that the antioxidant activity of all the tested samples was mostly related to their concentrations. The values of the scavenging effects ranged from 45 % to 96 % while the concentration of silymarin varied from 2 to 10 mg/mL. The result suggested that silymarin has a noticeable concentration-dependent effect on the scavenging of the hydroxyl radical.

#### Scavenging activity on superoxide radical

Mitochondria produce large amounts of superoxide anion (O_2_^2-^•) through the respiratory chain electron leakage pathways. The O_2_^2-^• is generated in the respiratory electron transport chain, by the one-electron reduction of oxygen, when electrons leak to the O_2_ molecule to form the highly reactive O_2_^2-^•. Such superoxide can further enhance the oxidation of H_2_O_2_ and form hydroxyl radical (•OH) and contribute to oxidative stress via the formation of reactive oxygen species. We measured the scavenging activity of the different silymarin extracts on the superoxide radicals by using the NADH-PMS-NBT system and ascorbic acid as control. The test results, shown in Figure 5c(Fig. 5) for the purified silymarin, indicated that the O_2_^2-^• radical scavenging increased from 25 % to 73 % while the concentration of purified silymarin increased from 0.2 to 2.0 mg/mL; and also showed that silymarin extract has a significant concentration-dependent antioxidant activity. 

#### Tyrosinase inhibitory activity

 TYR inhibitors are chemical agents capable of reducing enzymatic reactions, such as food browning and melanization of human skin. Therefore, these agents have commercial potential in the cosmetic industries. The result of the TYR inhibitory activity of purified silymarin is shown in Figure 5d(Fig. 5), and include data for Quercetin which was used as a control. The TYR inhibitory activity increased from 40 % to 70 % while the concentration of purified silymarin increased from 10 to 60 mg/mL. The results of the test system shows that silymarin extract has a significant concentration-dependent TYR inhibitory activity.

## Conclusion

In this study, the enzyme-assisted extraction of silymarin from the Silybum marianum seeds was investigated with a four-variable, three-level experiment Box-Behnken design based on RSM in order to optimize the silymarin extraction yield. In addition, the silymarin antioxidant activity was evaluated using a multi-test system in vitro. The results indicate that the enzyme-assisted method has advantages to process the seed shell silymarin-enrich extract, compared to the heating extraction from silymarin meal and it could be used as an effective method to extract silymarin from Silybum marianum. Another main finding of this work is the fact that the silymarin from Silybum marianum exhibited excellent antioxidant activity in the multi-test systems. 

Studies are in progress to further characterize the silymarin from Silybum marianum and elucidate their antioxidant mechanisms. Nevertheless, the knowledge gained from this study should be useful for further exploitation and application of the resource. On the basis of our results confirming the potent antioxidant properties of silymarin from Silybum marianum and previous findings about its metal chelator activity, the silymarin extract could be beneficial to humans as a health-promoting substance against oxidative stress; and thus useful to the antioxidant protection system in the food industry.

## Acknowledgements

The authors are grateful to the Analysis and Testing Center of Shenyang Agricultural University.

## Figures and Tables

**Table 1:**
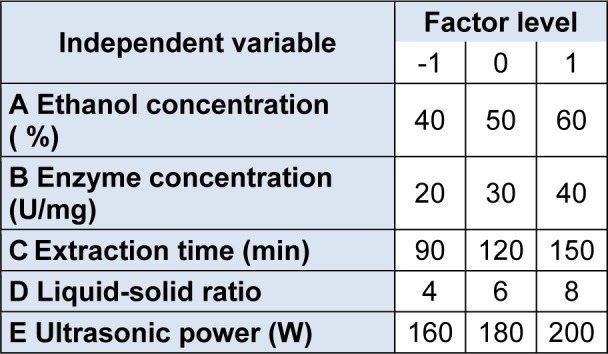
Independent variables and their levels used in the response surface design

**Table 2:**
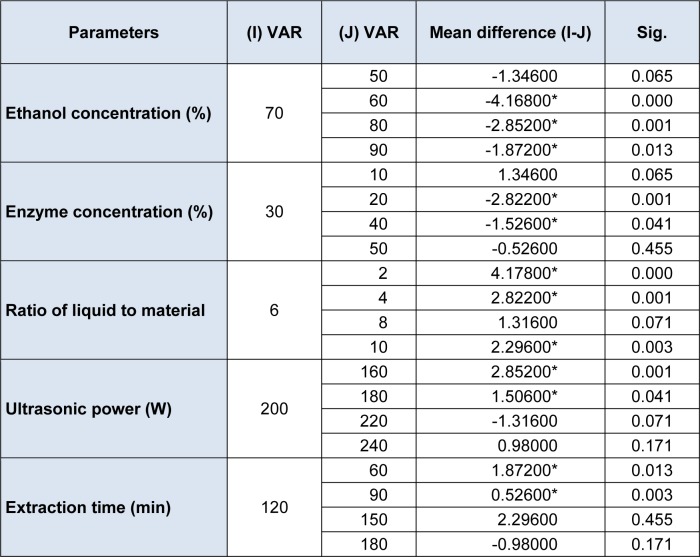
The results of multiple comparisons of the five parameters in silymarin extract

**Table 3:**
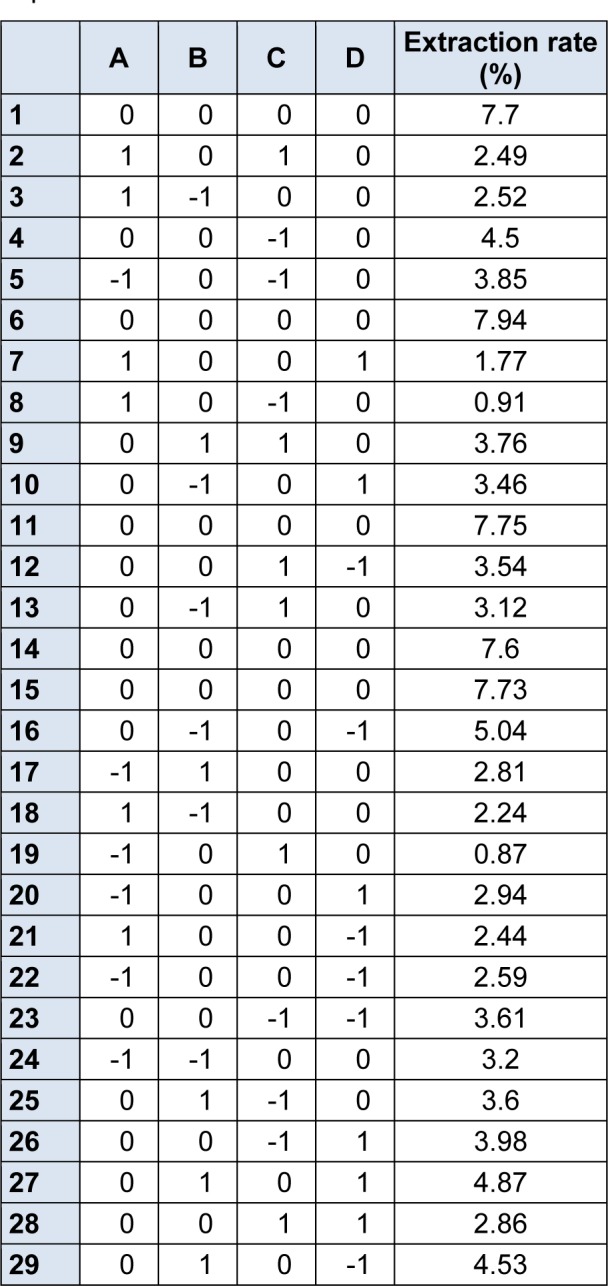
Factors and levels in the response surface central composite design arrangement and experimental results

**Table 4:**
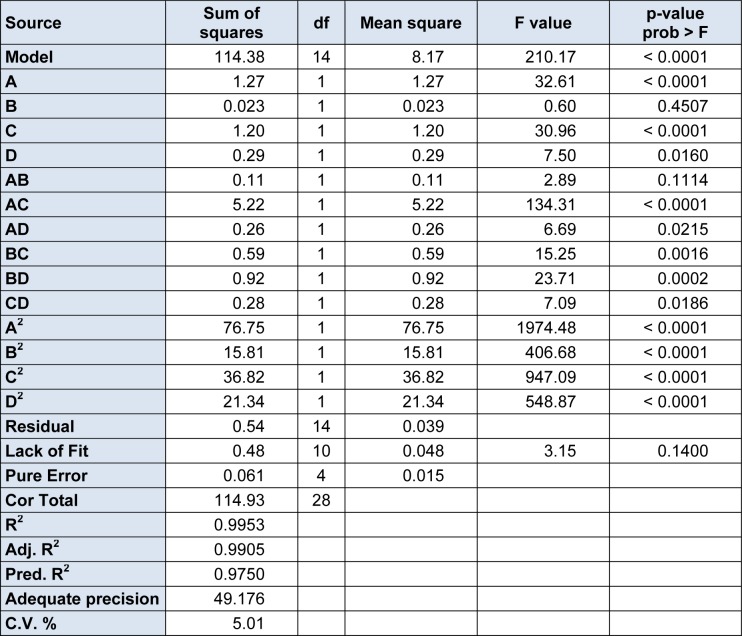
Analysis of variance (ANOVA) for the quadratic polynomial mode
